# High MELD 3.0 score is not an absolute contraindication to bariatric surgery

**DOI:** 10.1007/s00464-025-11940-w

**Published:** 2025-08-06

**Authors:** J. Alford Flippin, Shuqi Kang, Rana Omer Farman, Jasmin Patel, Faran Bokhari

**Affiliations:** 1 Trauma Surgery Department, OSF HealthCare Saint Francis Medical Center, 530 NE Glen Oak Avenue, North Bldg 3674, Peoria, IL 61637 USA; 2 University of Illinois College of Medicine, Peoria, USA

**Keywords:** MELD 3.0, Obesity associated liver disease, Preoperative risk assessment, Sleeve gastrectomy, Roux-en-Y gastric bypass

## Abstract

**Background:**

MELD 3.0 is a composite score used to predict mortality due to liver disease. Obesity is increasingly linked to liver disease and cirrhosis. Bariatric surgery is the most effective and durable method of managing morbid obesity and significantly improves many associated comorbid conditions, yet it is not without risk. Determining a reasonable risk profile for bariatric surgery is a complex decision made more difficult by lack of data regarding morbidity and mortality.

**Methods:**

We used data from the National Surgical Quality Improvement Program (NSQIP) from 2017–2019 to 2021–2022. Patients who underwent Roux-en-Y gastric bypass or sleeve gastrectomy were included. Each patient’s MELD 3.0 score was calculated. The primary outcome was mortality, calculated at each MELD 3.0 score. A line of best fit was constructed and used to calculate expected mortality rates for each MELD 3.0.

**Results:**

We identified 17,866 patients. Absolute observed mortality rates remained under 1% up to a MELD 3.0 of 18. Additionally, 167 patients with a MELD 3.0 greater than 20 were identified with only 1 mortality. The line of best fit showed an *r*^2^ value of 0.903 with predicted mortality remaining below 1% at a MELD 3.0 of 13, and below 4% at MELD 3.0 of 18. Due to insufficient data, the predictiveness of the model decreased above a MELD 3.0 of 19.

**Conclusion:**

Our data suggests that there are specific patients with seemingly prohibitive MELD 3.0 scores who are able to safely undergo bariatric surgery. A high MELD 3.0 score is not an absolute contraindication for bariatric surgery. Further research exploring safe patient selection criteria could allow bariatric surgeons to identify a group of patients that had previously been thought too high-risk for bariatric surgery, but who could benefit immensely from the weight loss and improvement in comorbid conditions that bariatric surgery provides.

The Model for End-Stage Liver Disease (MELD) score is a scoring system that was originally developed in 2001 as a method for using laboratory values to evaluate the severity of a patient’s chronic liver disease, specifically with regards to three-month survival to evaluate for candidacy for transjugular intrahepatic portosystemic shunt (TIPS) placement [1, 2]. Since its development, MELD has been adopted for a variety of applications, from prioritizing patients awaiting liver transplant to predicting survival in patients with alcohol-associated hepatitis [2]. Now in its third iteration, MELD 3.0 accounts for biologic sex, sodium bilirubin, albumin, International Normalized Ratio (INR), and creatinine [3]. Of late, MELD 3.0 has shown significant promise in its ability to risk stratify patients prior to nontransplant surgical intervention [1, 2]. Our research examines the ability of the MELD 3.0 scoring system to provide a method for risk stratifying bariatric patients.

In recent years, obesity has become a prominent source of liver disease. The obesity epidemic has been associated with a wide spectrum of liver disease, from simple steatosis to more advanced liver disease, such as nonalcoholic steatohepatitis (NASH), subsequent cirrhosis, and congestive hepatopathy from heart failure [4, 5]. Treatment primarily focuses on treatment of the underlying obesity. Although lifestyle modifications and pharmacotherapy are first line, bariatric surgery remains the treatment of choice when first line options fail [6].

Bariatric surgery has been shown to be the most effective and durable method of managing morbid obesity, but it is not without significant risk. Bariatric surgery aims to achieve dramatic weight loss as well as significantly improve and even eliminate many obesity-related comorbidities. An increasing number of studies demonstrate that bariatric surgery can reduce all cause death and cardiovascular mortality in bariatric patients. Bariatric surgery can also decrease rates of heart failure, stroke, and MI. It can vastly improve, if not completely resolve, conditions such as diabetes, sleep apnea, hypertension, hyperlipidemia, arthritis, and even fatty liver [6, 8, 10]. For patients with severe morbid obesity, who may not be able to exercise and fully participate in lifestyle modifications, bariatric surgery offers an opportunity to improve not only quantity, but also quality of life [10–12]. The patients with the highest BMIs and most severe medical comorbidities could potentially benefit the most from a bariatric procedure. Unfortunately, these patients are also often considered prohibitively high-risk candidates for surgery. In these cases, the decision to proceed with a bariatric procedure involves a complex discussion of risk profile and risk appetite.

Our research examines the utility of MELD 3.0 as a tool to facilitate discussions regarding a patient’s risk profile prior to bariatric surgery. We hypothesize that bariatric surgery may be safe for select patients at higher-than-expected MELD 3.0 scores.

## Materials and methods

Our research extracts data from the National Surgical Quality Improvement Program (NSQIP) from 2017–2019 and 2021–2022. We began by including all patients who underwent Roux-en-Y gastric bypass or sleeve gastrectomy. We excluded any patients with insufficient information to calculate a MELD 3.0 score. We excluded any surgeries that were revisions or conversions. We also excluded all emergent surgeries: all data included was derived from patients who underwent elective bariatric surgery. Each patient’s MELD 3.0 score was calculated. We looked at all-cause 30-day mortality as our primary outcome. For each MELD 3.0 score, the overall rate of 30-day mortality was calculated. Using this data, a line of best fit was constructed. For our secondary outcomes, we examined the patients who were still admitted or re-admitted at 30 days.

## Results

**Fig. 1:**
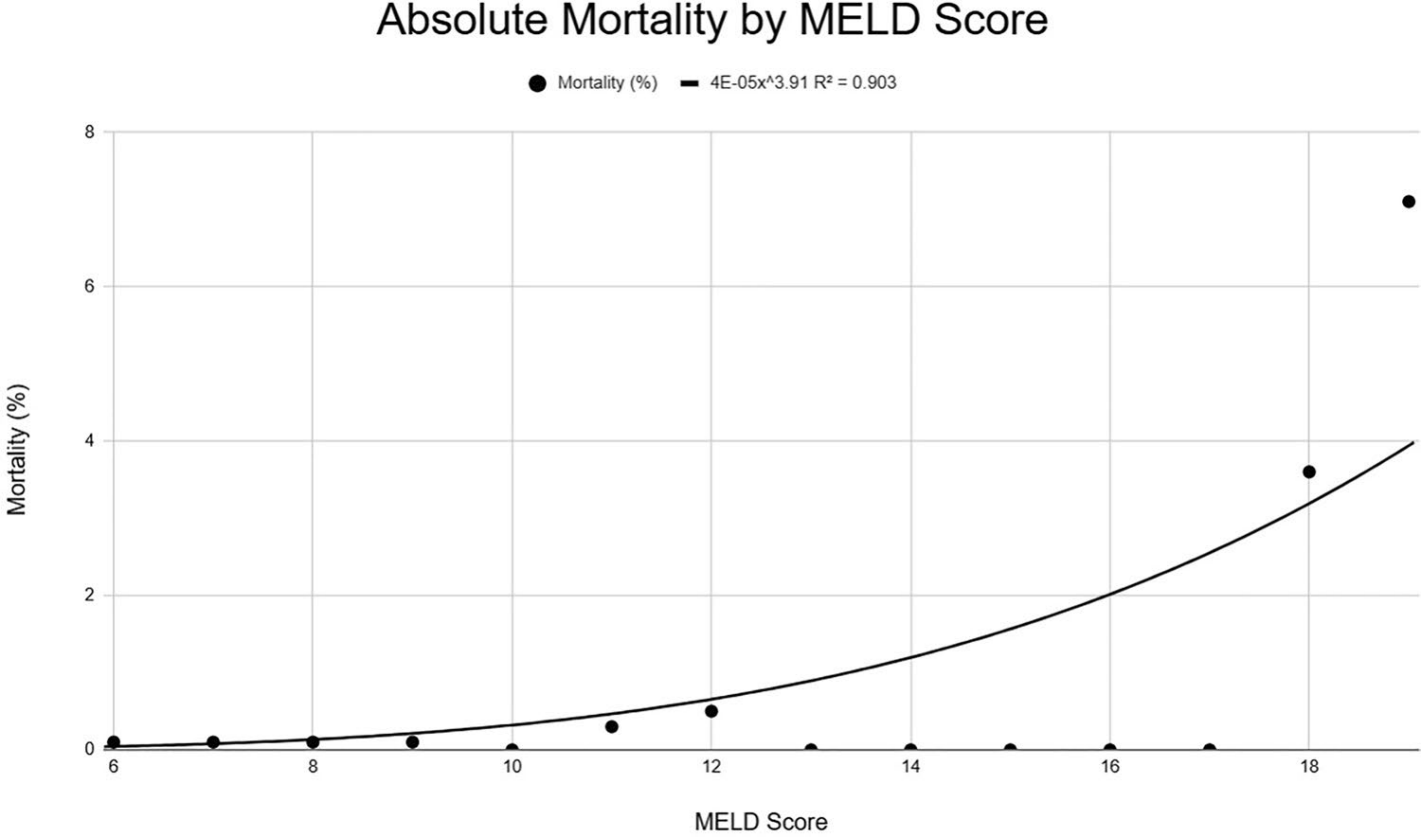
Line of best fit for mortality per MELD 3.0 score

We identified 17,866 patients with all necessary variables available to calculate corresponding MELD 3.0 scores and mortality rates. Up to a MELD 3.0 of 17, our absolute observed mortality rates remained under 1%. We identified 1683 patients with a MELD 3.0 of greater than or equal to 10 who underwent bariatric surgery, with only 5 mortalities—a 0.3% mortality rate. We identified 167 patients with a MELD 3.0 greater than 20 who underwent bariatric surgery, with only 1 mortality—a 0.6% mortality rate. Our projected line of best fit showed an r2 value of 0.903 and that mortality was predicted to remain below 1% at a MELD 3.0 of 13, and below 4% at MELD 3.0 of 18. Insufficiency of data precluded further exploration of predictive factors for mortality and the predictiveness of the model above a MELD 3.0 of 19 (Fig. 1 and Table 1).

**Table 1:** Mortality per MELD 3.0 score

MELD 3.0 score	Procedures	Mortality		
	*n* = 17866	*n* = 21	% (observed)	% (predicted)
6	1541	2	0.13	0.04
7	8419	8	0.09	0.08
8	4650	4	0.09	0.14
9	1568	2	0.13	0.22
10	613	0	0	0.33
11	315	1	0.32	0.47
12	182	1	0.55	0.66
13	137	0	0	0.9
14	91	0	0	1.21
15	55	0	0	1.59
16	52	0	0	2.04
17	34	0	0	2.59
18	28	1	3.57	3.24
19	14	1	7.14	4
20	14	0	0	4.89
21	22	0	0	5.91
22	18	0	0	7.09
23	49	0	0	8.44
24	24	0	0	9.97
25	14	0	0	11.7
26	3	0	0	13.6
27	4	0	0	15.8
28	12	0	0	18.2
29	3	0	0	20.9
30	1	0	0	23.9
31	2	1	50	27.1
32	1	0	0	30.7

As a secondary outcome, we examined 30-day re-admission rates. Of the 17,866 patients, only 9 patients either remained hospitalized at 30 days or were re-admitted at 30 days. Of these 9 patients, 3 had a MELD 3.0 score of 7 and 3 had a MELD 3.0 score of 8. The remaining 3 patients had a MELD 3.0 score of 9, 11, and 17. The patient with a MELD 3.0 of 17 also had a known bleeding disorder. Most of these patients were still admitted or re-admitted due to postoperative leak or bleed. 6 of these 9 patients required re-operation. Ultimately, none of these patients suffered mortality.

## Discussion

For any bariatric surgeon and bariatric patient, the question of risk versus benefit is an important one. For most bariatric centers of excellence, the accepted mortality rate for bariatric surgery is 0.1–0.2%. For patients with a significant burden of disease secondary to morbid obesity, bariatric surgery may seem too high-risk, despite the potential for life-changing benefits [7, 9, 12]. Our research was originally designed with the goal of evaluating the potential for MELD 3.0 score to assess preoperative risk in these seemingly high-risk patients. We had hypothesized that bariatric surgery may actually be relatively safe at higher-than-expected MELD 3.0 scores.

Our data shows that the observed mortality rate remained less than 1%, even on the higher end of MELD 3.0 scores. Bariatric surgery at a MELD 3.0 of greater than or equal to 10 carried a 0.3% mortality rate. Bariatric surgery at a MELD 3.0 score of greater than or equal to 20 carried a 0.6% mortality rate. Although our data becomes increasingly scarce at the higher MELD 3.0 scores, there is a surprising number of patients at these seemingly prohibitive MELD 3.0 scores who were able to safely undergo a potentially life-changing bariatric procedure. It is also important to note the difference between the predicted mortality rates generated by our line of best fit, and the actual observed mortality rates. As the MELD 3.0 scores increase, our actual observed mortality rates are consistently lower than those predicted by our line of best fit. Our results show that high MELD 3.0 bariatric patients do better than expected. While the reasons for this difference are unclear, we hypothesize that these high-MELD patients are being carefully selected in a limited number of centers. Identifying the criteria used to identify high-MELD patients in whom bariatric surgery can be safely performed is worth investigating as a an avenue for future research.

Our data suggests that a high MELD 3.0 score is not an absolute contraindication to bariatric surgery. In fact, for 167 carefully selected patients, bariatric surgery was a safe, potentially lifealtering treatment option. The results of our data aim to spark conversation and further research regarding the specific patient selection criteria and aspects of patient care that can make bariatric surgery feasible and safe in these high MELD 3.0 patients. While mortality rates of 0.3—0.6% are certainly higher than the 0.1–0.2% goals for bariatric surgery, the increased risk of mortality must also be weighed against the patient’s baseline mortality risk. Without correction of underlying comorbid conditions, some patients with severe morbid obesity may already have substantial short- and medium-term mortality risk. These factors would form the crux of the preoperative discussion regarding risks and benefits [11, 12].

There is an increasing number of patients for whom an opportunity for weight loss and improvement of obesity associated comorbid conditions would be lifesaving, but who might be thought of too high risk for bariatric surgery due to these comorbid conditions. Our data provides a challenge to these assumptions by demonstrating that there are specific bariatric patients at seemingly prohibitive MELD 3.0 scores who may be safe candidates for bariatric surgery. Further research could allow us to identify any as-yet uncharacterized variables that could guide candidacy for bariatric surgery for patients with severe morbid obesity.
